# *CsSWEET2*, a Hexose Transporter from Cucumber (*Cucumis sativus* L.), Affects Sugar Metabolism and Improves Cold Tolerance in Arabidopsis

**DOI:** 10.3390/ijms23073886

**Published:** 2022-03-31

**Authors:** Liping Hu, Feng Zhang, Shuhui Song, Xiaolu Yu, Yi Ren, Xuezhi Zhao, Huan Liu, Guangmin Liu, Yaqin Wang, Hongju He

**Affiliations:** 1 Institute of Agri-Food Processing and Nutrition, Beijing Academy of Agricultural and Forestry Sciences, Beijing 100097, China; huliping@iapn.org.cn (L.H.); songshuhui@iapn.org.cn (S.S.); yuxiaolu@iapn.org.cn (X.Y.); lysozyme@foxmail.com (X.Z.); huanl0329@163.com (H.L.); liuguangmin@iapn.org.cn (G.L.); 2 Beijing Key Laboratory of Fruits and Vegetable Storage and Processing, Beijing 100097, China; 3 Key Laboratory of Vegetable Postharvest Processing of Ministry of Agriculture and Rural Areas, Beijing 100097, China; 4 Beijing Vegetable Research Center, Beijing Academy of Agriculture and Forestry Sciences, Beijing 100097, China; zhangfeng@nercv.org (F.Z.); renyi@nercv.org (Y.R.)

**Keywords:** *CsSWEET2*, cucumber, hexose transporter, cold stress, plasma membrane, endoplasmic reticulum

## Abstract

Sugars, which are critical osmotic compounds and signalling molecules in plants, and Sugars Will Eventually be Exported Transporters (SWEETs), which constitute a novel family of sugar transporters, play central roles in plant responses to multiple abiotic stresses. In the present study, a member of the *SWEET* gene family from cucumber (*Cucumis sativus* L.), *CsSWEET2*, was identified and characterized. Histochemical analysis of β-glucuronidase expression in transgenic Arabidopsis plants showed that *CsSWEET2* is highly expressed in the leaves; subcellular localization indicated that CsSWEET2 proteins are localized in the plasma membrane and endoplasmic reticulum. Heterologous expression assays in yeast demonstrated that *CsSWEET2* encodes an energy-independent hexose/H^+^ uniporter that can complement both glucose and fructose transport deficiencies. Compared with wild-type Arabidopsis plants, transgenic Arabidopsis plants overexpressing *CsSWEET2* had much lower relative electrolyte leakage levels and were much more resistant to cold stress. Sugar content analysis showed that glucose and fructose levels in the transgenic Arabidopsis plants were significantly higher than those in the wild-type plants. Taken together, our results suggest that, by mediating sugar metabolism and compartmentation, *CsSWEET2* plays a vital role in improving plant cold tolerance.

## 1. Introduction

Sugars synthesized in leaf mesophyll cells are transported not only between different subcellular compartments but also between different cells to provide a long-distance carbon supply for sink organs (e.g., roots, seeds, and fruits) through sugar transporters [1,2]. To date, three classes of sugar transporters have been identified in plants: sucrose transporters/sucrose carriers (SUTs/SUCs), monosaccharide transporters (MSTs), and Sugars Will Eventually be Exported Transporters (SWEETs) [3]. Among these, SUTs/SUCs and MSTs generally have 12 α-helical transmembrane domains (TMs) and are members of the major facilitator superfamily, whereas SWEETs have only seven TMs and belong to the MtN3 family [3,4,5,6]. Constituting a unique class of sugar transporters, SWEETs have been identified as sugar uniporters that mediate both the influx and the efflux of hexose or sucrose across cell membranes along a sugar gradient and act as critical players in intracellular and intercellular sugar translocation [1,3,7,8].

Plants are often subjected to a variety of abiotic stresses (i.e., high salinity, flooding, drought, and extreme temperature) that adversely affect plant growth and development and cause massive losses in global crop yields [9]. Interestingly, recent studies have found that SWEETs play essential roles in plant stress resistance. In Arabidopsis, the expression of *AtSWEET15*, which is also named *SAG29*, is induced in response to drought, cold, and high salinity in an abscisic acid (ABA)-dependent manner, and *atsweet15* mutants exhibit reduced sensitivity to high salinity [10]. Similarly, rice *OsSWEET13* and *OsSWEET15* participate in the response to drought and salt stress by binding their promoters to the ABA-responsive transcription factor OsbZIP72 [11]. In addition, apple *MdSWEET17* plays an essential role in the plant’s resistance to drought [12]. The *atsweet11*/*12* double mutants accumulate more glucose, fructose, and sucrose and present much lower electrical conductivity than wild-type (WT) plants do under cold treatment, thereby improving the cold resistance of Arabidopsis [13]. Moreover, Arabidopsis transgenic plants overexpressing *AtSWEET4* or *AtSWEET16* show an altered sugar metabolism and are more tolerant of cold or drought [2,14]. In the tea plant, the expression of *CsSWEET16*, whose encoded protein is localized in the vacuolar membrane, is downregulated under cold stress, while the transcript levels of *CsSWEET1a* and *CsSWEET17*, both of whose proteins localize to the plasma membrane, significantly increase [15,16]. Remarkably, Arabidopsis transgenic plants overexpressing these transporters show increased tolerance to cold stress [15,16]. These results indicate that *CsSWEET1a* and *CsSWEET17* may adopt a pathway different from that used by *CsSWEET16* to protect plants under cold stress. In *Dianthus spiculifolius*, the transcript levels of *DsSWEET12* and *DsSWEET17* increase under salt, osmotic, and oxidation stresses [17,18]. Overexpression of *DsSWEET12* or *DsSWEET17* in Arabidopsis seedlings alters the sugar metabolism, thereby improving resistance to various stresses [17,18]. These studies clearly demonstrate that SWEETs have the potential to improve plant performance and yield under adverse environmental conditions by regulating sugar transport and distribution. Therefore, SWEETs need to be studied in more detail and examined in-depth in more species in order to improve the survival and yield potential of said species under a range of challenging environmental conditions.

Cucumber (*Cucumis sativus* L.), one of the most important vegetable crop species worldwide, is sensitive to various abiotic stresses, including salinity, drought, and cold, which cause significant yield and economic losses. Owing to its tropical origin and heat-loving habit, cucumber is extremely sensitive to cold stress. In previous studies, 17 *CsSWEET* genes were identified in the cucumber genome by bioinformatic analysis [19,20], among which are *CsSWEET7a*, responsible for sugar phloem unloading in cucumber fruits, receptacles, and nectaries [21,22], and *CsSWEET12c*, which is involved in promoting plant growth and flowering [23]. However, the exact roles of the other 15 *CsSWEET* genes in cucumber have not yet been established. Furthermore, the functions of the *CsSWEET* genes in cucumber in response to abiotic stresses are poorly understood. In this study, we cloned *CsSWEET2* from cucumber and studied its tissue-specific expression patterns and the substrate specificities and cellular localization of its encoded protein. Moreover, we generated *CsSWEET2* overexpression transgenic Arabidopsis lines and analysed the contribution of *CsSWEET2* to cold resistance. These results contribute to improving the cold tolerance of cucumber through molecular breeding techniques.

## 2. Results

### 2.1. CsSWEET2 Encodes a Clade I SWEET Protein

**Figure 1:**
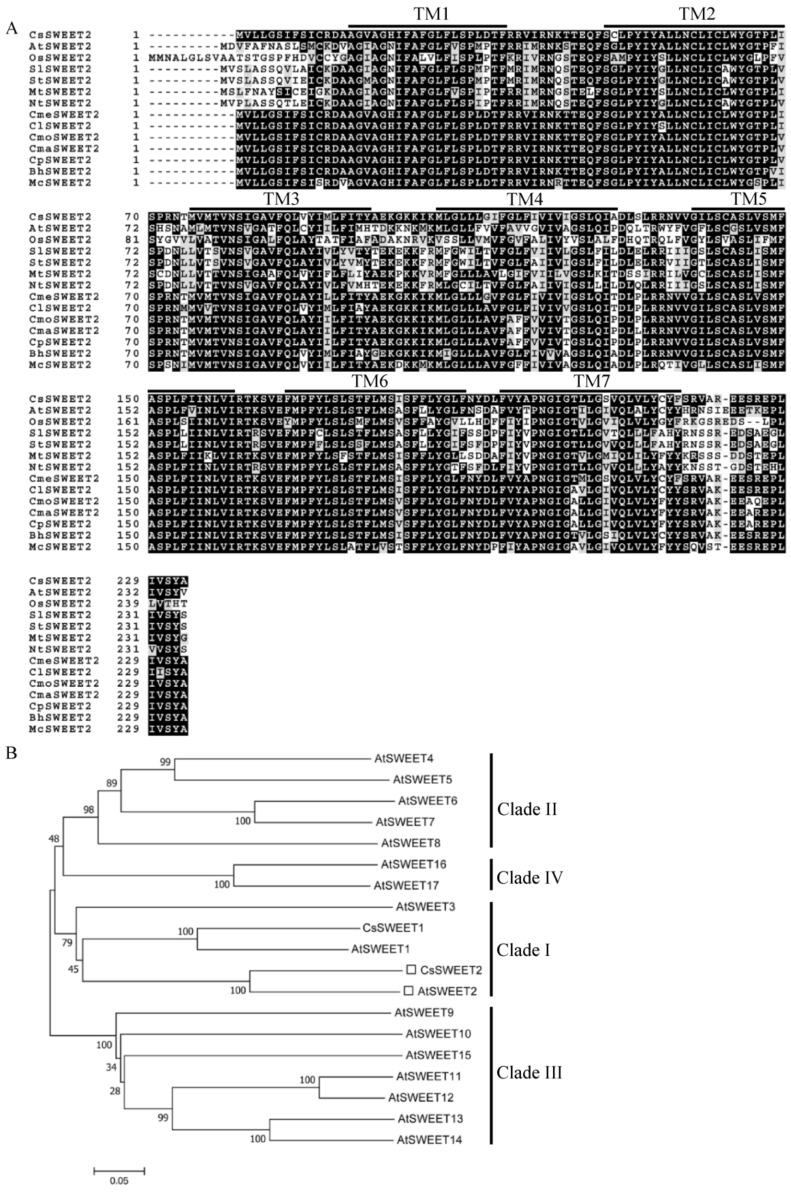
Sequence analysis of CsSWEET2. (**A**) Multiple sequence alignment of SWEET2 proteins from Cucumis sativus (CsSWEET2), Arabidopsis thaliana (AtSWEET2), Oryza sativa (OsSWEET2), Solanum lycopersicum (SlSWEET2), Solanum tuberosum (StSWEET2), Medicago truncatula (MtSWEET2), Nicotiana tabacum (NtSWEET2), Cucumis melo (CmeSWEET2), Citrullus lanatus (ClSWEET2), Cucurbita moschata (CmoSWEET2), Cucurbita maxima (CmaSWEET2), Cucurbita pepo (CpSWEET2), Benincasa hispida (BhSWEET2), and Momordica charantia (McSWEET2). The seven transmembrane domains (TMs) are outlined. The conserved amino acids are indicated by the grey background, and identical amino acids are denoted by the dark shading. (**B**) Phylogenetic analysis of SWEET proteins from cucumber (CsSWEET2) and Arabidopsis (AtSWEET1 to AtSWEET17). The amino acid sequences of the SWEET proteins used for the analysis are listed in Table S3.

A putative *SWEET* gene was cloned from the total RNA of cucumber leaves and named *CsSWEET2* (GenBank ID: MG004672) based on its closest phylogenetic relationship with *AtSWEET2* from Arabidopsis. The cloned 1324 bp full-length *CsSWEET2* cDNA comprises a 702 bp open reading frame (ORF), a 319 bp 5′-untranslated region, and a 303 bp 3′-untranslated region (Table S1). *CsSWEET2* encodes a polypeptide of 233 amino acids with a molecular weight of 26.07 kDa, an isoelectric point of 9.18, and a grand average hydropathicity of 0.932 (Table S1). In addition, the distribution of exons and introns within the *CsSWEET2* gene was analysed to determine the gene structure. The genomic sequence of *CsSWEET2* is 2503 bp long and comprises six exons and five introns (Supplementary Figure S1A). Typical plant SWEET proteins include seven TMs containing two conserved MtN3/saliva motifs [3,4,5,6]. A search of the CsSWEET2 protein sequence via a MOTIF Search (https://www.genome.jp/tools/motif) revealed two conserved MtN3/saliva domains (Supplementary Figure S1B). Additionally, CsSWEET2 was predicted via TMHMM analysis to have seven TMs (Supplementary Figure S1C). These data, together with multiple sequence alignment results (Figure 1A), show that the CsSWEET2 protein is a typical 7-TM domain-containing SWEET. According to phylogenetic analysis, CsSWEET2 is most closely related to AtSWEET2 (69.07% amino acid sequence identity) (Figure 1B; Table S2) and belongs to clade I of the SWEET family [7]. Moreover, the amino acid identity of CsSWEET2 was highly similar to those of SWEET2 proteins from other plant species (Figure 1A; Table S2).

### 2.2. Expression Analysis of CsSWEET2 in Different Tissues

**Figure 2:**
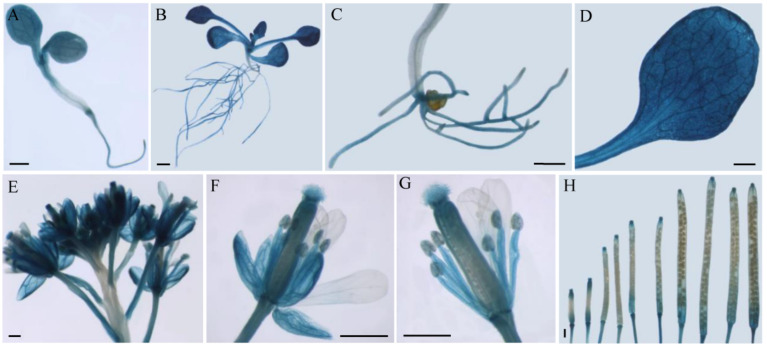
Spatial expression patterns of *CsSWEET2* in transgenic Arabidopsis plants. (**A**) Seven-day-old seedling. (**B**,**C**) Two-week-old seedling. (**D**) Rosette leaf. (**E**) Inflorescence. (**F**,**G**) Flowers at higher magnification showing β-glucuronidase (GUS) staining in the sepals (**F**), filaments (**G**), and pistils (**F**,**G**). (**H**) Siliques at different stages. Scale bars = 1 mm.

In our previous study, the transcription profile of various tissues of three-month-old cucumber plants (leaves, stems, roots, male flowers, fruits, and tendrils) was analysed via quantitative real-time PCR (qRT-PCR) to determine the expression patterns of 17 *CsSWEET* genes [19]. The results revealed that *CsSWEET2* was mainly expressed in cucumber leaves, roots, male flowers, and fruits [19]. To obtain detailed expression profiles at the tissue level during plant development, we generated transgenic Arabidopsis plants expressing the *β*-*glucuronidase* (*GUS*) gene under the control of the *CsSWEET2* promoter. In the *pCsSWEET2::GUS* transgenic plants, GUS activity was consistently high in all the leaf and root samples but not in the hypocotyl samples (Figure 2A–D). In addition, GUS activity was present within all the mesophyll tissues of the cotyledons, young leaves, and rosette leaves (Figure 2A,B,D). At the flowering stage, GUS activity was high in the peduncles and all developing flower buds (Figure 2E). Further investigation revealed that the expression was localized to the sepals, pistils (mainly in stigma/style), and filaments (Figure 2F,G). In contrast, the petals, anthers, and pollen did not exhibit any GUS activity (Figure 2F,G). Moreover, GUS activity was seemingly high at both ends of the siliques at every stage of silique development but not in the seeds (Figure 2H). Overall, the results of GUS staining in Arabidopsis were similar to the tissue expression pattern analysed via qRT-PCR in cucumber plants [19].

### 2.3. CsSWEET2 Is Localized to the Plasma Membrane and Endoplasmic Reticulum

**Figure 3:**
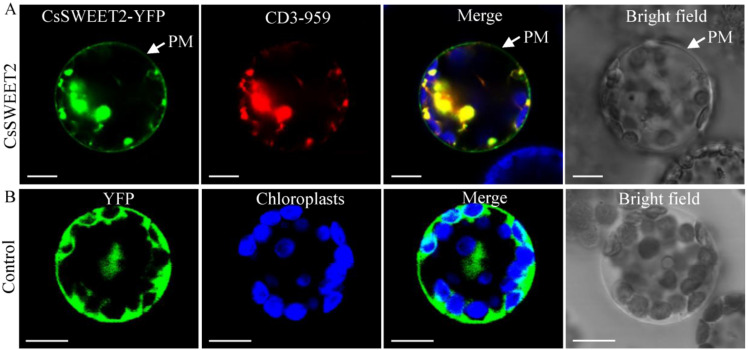
Subcellular localization of the CsSWEET2-YFP fusion protein in Arabidopsis mesophyll protoplasts. An mCherry-labelled marker (CD3-959) was used to mark the endoplasmic reticulum position. The arrows in (**A**) point to plasma membranes. An empty vector expressing untargeted YFP was used as a control (**B**). The green signals indicate YFP, whereas the red signals indicate the endoplasmic reticulum marker. In addition, the blue signals indicate the chlorophyll auto fluorescence. Their merged images and bright field images are also presented. PM, plasma membrane. Scale bars = 10 μm.

The subcellular localization of CsSWEET2 was first analysed through the transient expression of CsSWEET2-YFP fusion proteins in cucumber mesophyll protoplasts. Fluorescence signals derived from CsSWEET2-YFP fusion proteins were observed not only at the plasma membrane but also in the reticular formation in cucumber protoplasts (Supplementary Figure S2A). The same results were observed in the chloroplast-free protoplasts of etiolated rice (Supplementary Figure S2B). Based on these results, we speculated that CsSWEET2 likely localizes to both the plasma membrane and the endoplasmic reticulum. To confirm this possibility, an mCherry-labelled endoplasmic reticulum marker was cotransformed with CsSWEET2-YFP into protoplasts of cucumber or Arabidopsis. The cucumber protoplasts failed to coexpress the endoplasmic reticulum marker and CsSWEET2-YFP despite many attempts, but the Arabidopsis protoplasts succeeded in doing so. As shown in Figure 3A, the green fluorescence emitted by CsSWEET2-YFP in the reticular formation is coincident with the red fluorescence of the endoplasmic reticulum marker. These results strongly indicate that CsSWEET2 functions predominantly at the plasma membrane and the endoplasmic reticulum (Figure 3A), whereas the YFP control vector exhibited fluorescence throughout whole cells (Figure 3B).

### 2.4. CsSWEET2 Transports Glucose and Fructose in Yeast

**Figure 4:**
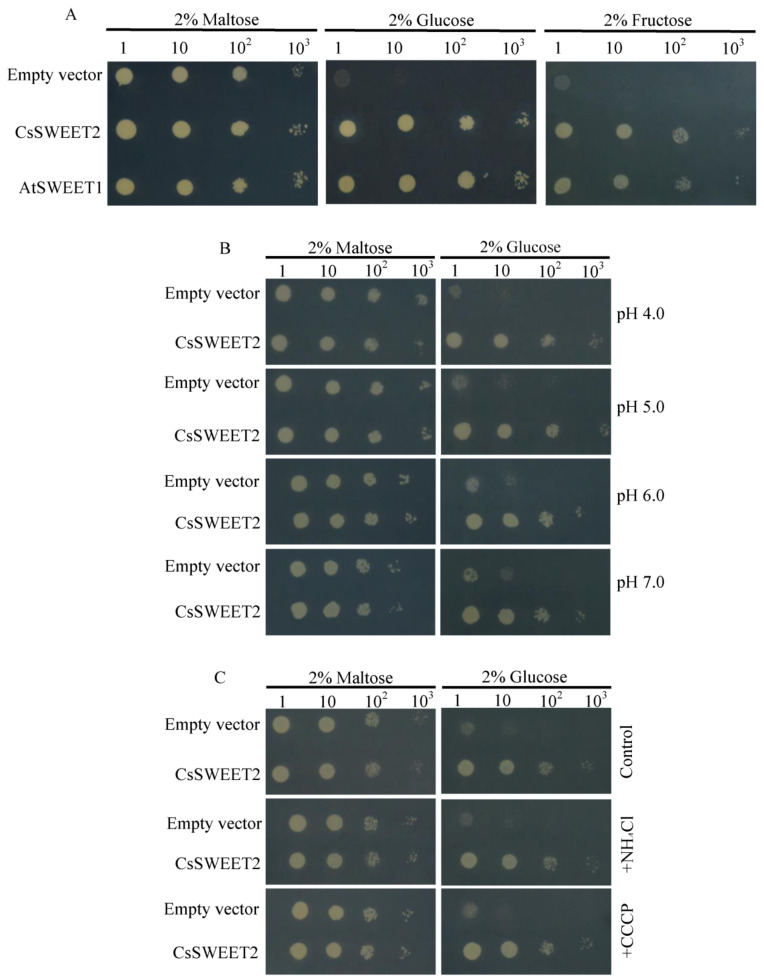
Sugar transport activities of *CsSWEET2* expressed in the yeast mutant EBY.VM4000. (**A**) *CsSWEET2* and *AtSWEET1* complemented the glucose and fructose uptake deficiency of EBY.VM4000, but the empty vector did not. (**B**) Functional complementation of the glucose uptake deficiency of EBY.VM4000 by *CsSWEET2* at different pHs. (**C**) Effects of metabolic inhibitors on yeast growth.Yeast cells expressing an empty vector or a vector containing *CsSWEET2* or *AtSWEET1* were serially diluted (10-fold) and cultured on selective synthetic deficient media without uracil (SD-Ura) supplemented with 2% (*w/v*) maltose, 2% (*w/v*) glucose, or 2% (*w/v*) fructose as the sole carbon source. Images were captured after incubation at 30 °C for 3 days. An empty vector (pDRf1-GW) was used as a negative control. *AtSWEET1*, which has been shown to mediate glucose and fructose uptake in the yeast EBY.VM4000 mutant [25], was used as a positive control. CCCP, carbonyl cyanide m-chlorophenyl hydrazine.

To investigate the transport properties of *CsSWEET2*, the gene was expressed in the hexose-uptake-deficient yeast (*Saccharomyces cerevisiae*) mutant EBY.VM4000, which cannot grow on media that include hexose but can grow on media that include maltose [24]. Complementation growth assays showed that yeast expressing *CsSWEET2* could grow on media that include glucose or fructose (Figure 4A). In contrast, the yeast transformed with the empty pDRf1-GW vector (as a control) grew very poorly on the media, including glucose or fructose (Figure 4A). These results indicate that *CsSWEET2* encodes a functional hexose transporter.

To identify whether *CsSWEET2* hexose transport is pH dependent, we tested the glucose uptake using EBY.VM4000 with *CsSWEET2* expression in media supplemented with 2% (*w/v*) maltose or 2% (*w/v*) glucose at pH 4.0, 5.0, 6.0, and 7.0. The transformants showed similar trends at each pH (Figure 4B), suggesting that the transport activity of *CsSWEET2* is not dependent on pH. Moreover, treatment with the uncoupler NH_4_Cl or the protonophore carbonyl cyanide m-chlorophenyl hydrazine (CCCP), which disrupts the pre-existing proton gradient, did not significantly inhibit the growth of yeast cells expressing *CsSWEET2* in maltose- or glucose-supplemented media (Figure 4C), indicating that *CsSWEET2* functions as an energy-independent facilitative transporter.

### 2.5. Expression of CsSWEET2 in Response to Abiotic Stresses

**Figure 5:**
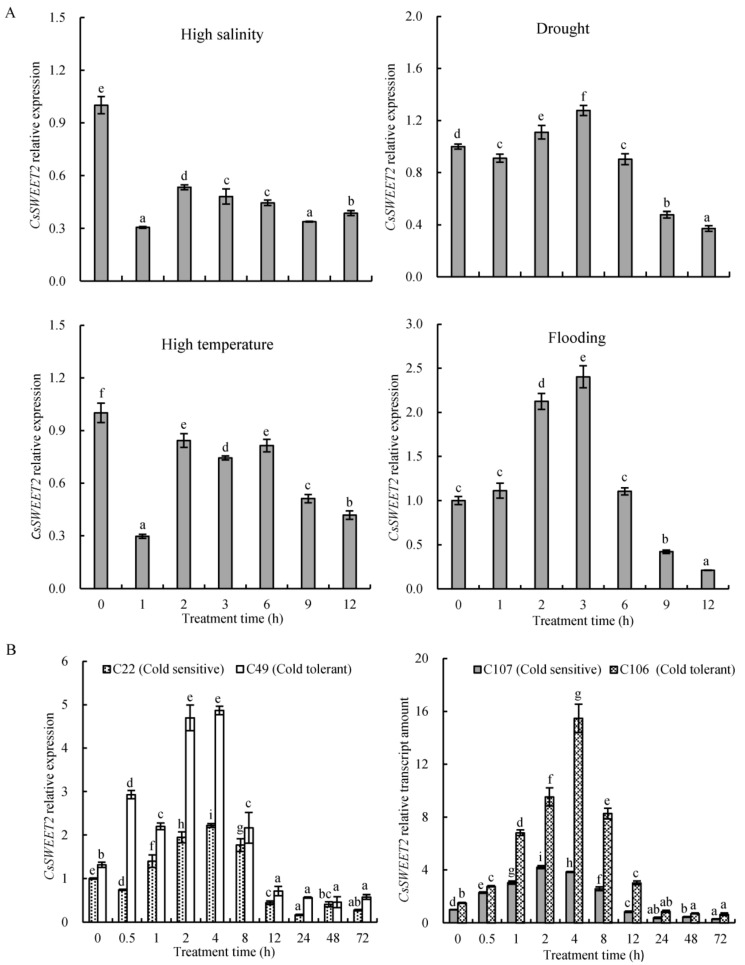
Expression profiles of *CsSWEET2* in the leaves of cucumber seedlings under abiotic stresses. (**A**) Seedlings (inbred line C49) at the 3–4 true leaf stage were used for subsequent treatments involving high salinity (200 mM NaCl), drought (10% polyethylene glycol 6000), high temperature (40 °C), and flooding. (**B**) Seedlings (inbred lines C22, C49, C106, and C107) at the 3–4 true leaf stage were used for subsequent cold-stress (10 °C) treatment. The relative expression levels of *CsSWEET2* were detected via quantitative real-time PCR (qRT-PCR), and the cucumber *actin* gene was used as a reference for normalization of the expression data. The data are presented as the means ± SDs of three replicates. The different letters above the bars indicate significant differences (*p* < 0.05).

Since SWEETs play critical roles in plant responses to multiple abiotic stresses [26], we were interested in analysing whether *CsSWEET2* also contributes to abiotic stress tolerance. Cucumber seedlings at the 3–4 true leaf stage were subjected to various abiotic stress treatments, and the time course changes in mRNA levels of *CsSWEET2* expressed in the leaves were analysed by qRT-PCR. Under high-salinity treatment, *CsSWEET2* expression decreased significantly at 1 h and then recovered somewhat, although it remained at a low level (Figure 5A); similarly, the expression was also reduced under high temperature, although to a lesser degree (Figure 5A). In contrast, the transcript level of *CsSWEET2* increased, peaked at 3 h, and then declined during drought or flooding treatment (Figure 5A).

As cucumber easily succumbs to cold injury in regions such as northern China during the winter and early spring, we paid particular attention to the expression profile of *CsSWEET2* under cold treatment. The seedlings of two inbred lines of northern China-type cucumbers, specifically C22 (a cold-sensitive line) and C49 (a cold-tolerant line), and two inbred lines of Europe-type cucumbers, specifically C107 (a cold-sensitive line) and C106 (a cold-tolerant line), were exposed to cold treatment (10 °C). As shown in Figure 5B, the *CsSWEET2* transcript levels of all four inbred lines increased significantly, peaked at 2 h or 4 h, and then declined. Notably, *CsSWEET2* expression was consistently higher in the cold-tolerant lines than in the cold-sensitive lines during cold treatment (Figure 5B). These results prompted us to further investigate the function of *CsSWEET2* in response to cold stress.

### 2.6. Overexpression of CsSWEET2 in Arabidopsis Improves Tolerance to Cold Stress

**Figure 6:**
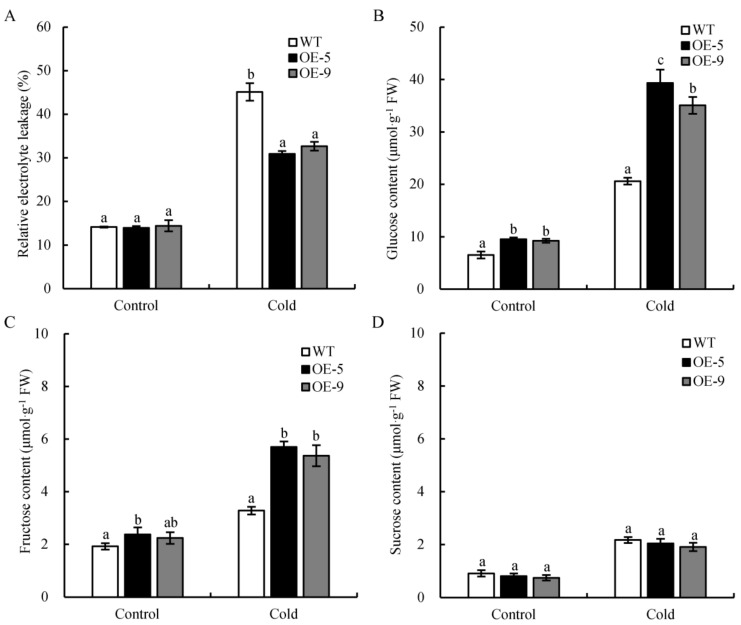
Analysis of the relative electrolyte leakage (REL) levels and sugar contents in *CsSWEET2*-overexpressing (*CsSWEET2*-OE) lines and wild-type (WT) plants. The four-week-old plants were grown at 4 °C for 4 days for cold-stress treatment, after which the whole shoots were collected for measurements of REL and sugar contents. (**A**) REL levels. (**B**–**D**) Contents of glucose (**B**), fructose (**C**), and sucrose (**D**). The data are presented as the means ± SDs of three replicates. The different letters above the bars indicate significant differences (*p* < 0.05).

To determine whether increased *CsSWEET2* expression improves cold tolerance, transgenic Arabidopsis lines overexpressing *CsSWEET2* driven by the cauliflower mosaic virus (CaMV) 35S promoter were generated. Among the resulting 12 strong *CsSWEET2-*overexpressing (*CsSWEET2*-OE) lines, lines 5 and 9 (OE-5 and OE-9), which presented much higher *CsSWEET2* transcript levels than the WT plants did and whose *AtSWEET2* expression was not affected, were selected to generate homozygous lines for further experiments (Supplementary Figure S3). The WT plants and T_3_ homozygous of OE-5 and OE-9 transgenic lines were grown for 4 weeks under normal conditions (22 °C) and then transferred to cold treatment (4 °C) for 4 days. Whole shoots were removed to quantify the release of electrolytes, which serves as an indicator of how many cells have been destroyed [27]. Under normal conditions, the relative electrolyte leakage (REL) levels of OE-5 and OE-9 lines showed no significant differences compared with those of WT. After cold treatment, the REL levels of the two OE lines were significantly lower than those of WT plants (Figure 6A), which indicated that the *CsSWEET2*-OE lines were less damaged by cold stress and that an increase in *CsSWEET2* levels improved the cold tolerance of Arabidopsis.

Given that *CsSWEET2* can transport glucose and fructose (Figure 4A) and that changes in cold tolerance may result from changes in sugar contents, we compared the sugar contents between the *CsSWEET2*-OE and WT plants. As shown in Figure 6B,C, the two OE lines accumulated significantly more glucose and fructose than the WT plants did when exposed to cold treatment. In addition, the *CsSWEET2*-OE and WT plants did not show significant differences in sucrose contents (Figure 6D). Taken together, these results indicate that an increase in *CsSWEET2* expression contributes to the accumulation of glucose and fructose and results in improved cold tolerance of Arabidopsis.

## 3. Discussion

In line with the rapid growth of SWEETs research in Arabidopsis [2,13,14,28,29], rice [11], and maize [30,31,32], studies on *SWEET* homologues in other plant species such as tomato [33], watermelon [25], and the tea plant [15,16] have been reported, and findings have been updated. Although SWEETs are important versatile regulators and affect yield potential, only two cucumber *CsSWEET* genes have been reported in detail to date [21,22,23]. In this study, we characterized the *CsSWEET2* gene in cucumber and found that overexpression of *CsSWEET2* in Arabidopsis improves tolerance to cold stress.

### 3.1. CsSWEET2 Is a Glucose and Fructose Transporter Localized to the Plasma Membrane and Endoplasmic Reticulum

The class of sugar transporters named SWEETs was first identified by Chen et al. [7] in Arabidopsis, which contains 17 members categorized into four phylogenetic clades. In detail, clade I consists of *AtSWEET1* to *AtSWEET3*, clade II consists of *AtSWEET4* to *AtSWEET8*, clade III consists of *AtSWEET9* to *AtSWEET15*, and clade IV consists of *AtSWEET16* and *AtSWEET17*. Here, we cloned a member of the *SWEET* gene family from cucumber, *CsSWEET2*, for the first time and showed that *CsSWEET2* belongs to clade I and is phylogenetically most closely related to *AtSWEET2* (Figure 1). In Arabidopsis, *AtSWEET2* encodes a glucose transporter that is located on the vacuolar membrane, and its activity determines the glucose content of root vacuoles [34]. Like *AtSWEET2*, rice *OsSWEET2b*, which belongs to clade I, has been shown to localize to the vacuolar membrane and transport glucose [5]. In contrast to *AtSWEET2* and *OsSWEET2b*, *AtSWEET1* and tomato *SlSWEET1* (another two members of clade I) are localized on the plasma membrane [7,33]. AtSWEET1 is a glucose and fructose transporter that is highly expressed in flowers and may supply nutrients to gametophytes or nectaries [7,25], whereas *SlSWEET1* is a glucose transporter and may participate in glucose unloading in young leaves of tomato [33]. Overall, SWEETs from clade I preferentially transport hexose compounds, mainly glucose. In addition, except for *PagSWEET2c* and *PagSWEET3b*, which are localized in the endoplasmic reticulum in cells of *Populus* [35], they have been reported to be localized on the vacuolar membrane or plasma membrane. In the present study, our heterologous expression assay showed that *CsSWEET2* functions in both glucose and fructose transport rather than sucrose transport and can restore EBY.VW4000 growth on media supplemented with glucose or fructose (Figure 4A). Furthermore, glucose uptake by EBY.VW4000 transformed with *CsSWEET2* indicated that the transformants grew well in media with different pH values or that were supplemented with NH_4_Cl or CCCP (Figure 4B,C), proving that *CsSWEET2* is an energy-independent hexose/H^+^ uniporter. These data are consistent with the results from other *SWEET* gene members belonging to clade I, such as *AtSWEET1* [7,25].

In addition to transporting substrates, the functions of SWEETs largely depend on their subcellular localization. In our previous study, CsSWEET2 was predicted via WoLF PSORT (a bioinformatics tool for localization predictions) to localize to the nucleus [19]. However, in the present study, by expressing CsSWEET2-YFP fusion proteins in protoplasts of cucumber, rice, or Arabidopsis, we found that CsSWEET2 was localized in the plasma membrane and endoplasmic reticulum (Figure 3 and Figure S2). These results contrasted with the localization predictions of WoLF PSORT. The subcellular localization features of *CsSWEET2* are also different from those of other *SWEET* gene members of clade I reported thus far [5,7,33,34,35], including *AtSWEET2*, which is the closest homologue of *CsSWEET2* in Arabidopsis; thus, their functions could also be different.

### 3.2. CsSWEET2 Is Highly Expressed in the Leaves and Positively Correlated with Cucumber Cold Tolerance

It is well known that the physiological roles of sugar transporters are closely related to their tissue-specific expression. In previous studies, qRT-PCR analysis showed that, compared with other cucumber *SWEET* gene transcripts, *CsSWEET2* transcripts were high in mature leaves of cucumber [19,20]. Additionally, RNA sequencing data showed that *CsSWEET2* was one of the most highly expressed *CsSWEET* genes in cucumber leaves at various developmental stages, including cotyledons, first true leaves, young leaves, and mature leaves (Supplementary Figure S4). Furthermore, high activity of GUS driven by the *CsSWEET2* promoter was found in the mesophyll tissues of cotyledons, young leaves, and rosette leaves of transgenic Arabidopsis plants in the present study (Figure 2A,B,D). Taken together, these results confirm that, compared with other *CsSWEETs*, *CsSWEET2* is highly expressed in the mesophyll tissues of cucumber leaves at different developmental stages and probably plays vital roles during cucumber plant development. It has been reported that some SWEETs play crucial roles in mediating phloem loading or unloading in leaves; both processes are essential for sugar mobilization and may affect photosynthesis efficiency. In Arabidopsis, *AtSWEET11* and *AtSWEET12* are localized to the plasma membrane of cells composing leaf vascular tissue and mediate sucrose export from mesophyll cells to the apoplast [28]. Similarly, *ZmSWEET13a*, *ZmSWEET13b*, and *ZmSWEET13c*, which are specifically expressed in leaf vasculature, are involved in sucrose apoplastic phloem loading in maize [30,31]. In tomato, *SlSWEET1a*, which is highly expressed in the veins of young leaves, is localized to the plasma membrane and functions in glucose efflux from mature to young leaves [33]. However, since *CsSWEET2* is highly expressed in mesophyll tissues rather than veins (Figure 2A,B,D), we speculate that *CsSWEET2* may not participate in phloem loading in mature leaves or phloem unloading in young leaves.

SWEET-mediated sugar transport is essential for resistance to various abiotic stresses, such as high salinity, drought, or cold [2,10,11,13,14,15,16,17,18,26]. Thus, we speculated whether *CsSWEET2* plays a role in the stress response in cucumber and monitored its expression in response to high salinity, drought, high temperature, flooding, and cold. Interestingly, the expression of *CsSWEET2* in cold-tolerant lines was always higher than in cold-sensitive lines during cold treatment (Figure 5B). This phenomenon was observed not only in the northern China-type lines but also in the Europe-type lines (Figure 5B). Furthermore, the *CsSWEET2*-OE lines exhibited improved tolerance to cold stress, which was revealed by the significantly reduced REL levels (Figure 6A). Based on our results, we suggest that the increased expression of *CsSWEET2* contributes to the improved cold tolerance of plants.

### 3.3. Possible Function of CsSWEET2 in Enhancing Cucumber Cold Tolerance

Increasing evidence indicates that the accumulation of high levels of soluble sugars, which can act as osmoprotectants, helps plants tolerate cold stress [36,37]. In cucumber, cold treatment induced a significant accumulation of soluble sugars in various subcellular compartments in the leaves [38]. Specifically, it has been shown that galactinol accumulates in the cytosol and vacuoles; sucrose and raffinose accumulate in the cytosol, vacuoles, and chloroplasts; and stachyose accumulates in the vacuoles [38]. In addition to these soluble sugars, glucose and fructose contents were shown to increase in the leaves of cucumber plants subjected to cold treatment [39]. In the present study, the *CsSWEET2*-OE plants, whose glucose and fructose contents were significantly higher than the WT plants, showed higher resistance to cold stress (Figure 6A–C). This phenomenon has been observed in previous studies, which have shown a positive correlation between hexose content and tolerance to abiotic stress such as cold stress [14,16,17,18]. Moreover, as the key sugar transporters that mediate sugar transport and distribution, SWEETs are closely linked to sugar homeostasis within cellular compartments, which is vital for plant stress tolerance. In Arabidopsis, tonoplast-localized *AtSWEET16* is responsible for exporting glucose, fructose, and sucrose from the vacuole to the cytosol to adjust osmotic homeostasis and survive under cold stress [2]. In the tea plant, *CsSWEET16*, a homologue of *AtSWEET16*, is another vacuolar-membrane-localized sugar transporter that enhances cold tolerance by promoting fructose compartmentation in the vacuoles of transgenic Arabidopsis plants [15]. In general, cold stress first affects the vascular system and, subsequently, the mesophyll tissue [40]. Plasma-membrane-localized *CsSWEET1a* and *CsSWEET17* are mainly expressed in veins and mediate the partitioning of glucose and fructose between the apoplast and the cytosol, thereby increasing the cold tolerance of the tea plant [16]. In the present study, based on the characteristics of *CsSWEET2* in terms of its subcellular localization and substrates transported (Figure 3 and Figure 4), we speculated that *CsSWEET2* could mediate the uptake of glucose and fructose across the plasma membrane and efflux these sugars into the endoplasmic reticulum; that is, *CsSWEET2* can mediate the compartmentation and homeostasis of glucose and fructose among the apoplast, cytosol, and endoplasmic reticulum. A higher expression of *CsSWEET2* resulted in the transport of more glucose and fructose from the apoplast to the cytosol and endoplasmic reticulum along a sugar gradient, increasing the intracellular solute content and thus protecting cells from cold injury. Moreover, under conditions of oxidative stress such as cold, soluble sugars can serve as osmoprotectants or as signalling molecules to promote reactive oxygen species scavenging [36,37,39,41]. Therefore, overexpression of *CsSWEET2* can improve the cold tolerance of transgenic Arabidopsis plants (Figure 6).

Recent studies have demonstrated that *AtSWEET11* and *AtSWEET12* are directly phosphorylated by SnRK2 protein kinases, leading to enhanced drought resistance of Arabidopsis plants [29]. In rice, the expression of *OsSWEET13* and *OsSWEET15* is activated by the direct binding of their promoters to *OsbZIP72*, an ABA-responsive transcription factor, resulting in the maintenance of sugar homeostasis and improved resistance to drought and salinity stresses [11]. Using bioinformatics software/tools, we identified not only two possible phosphorylation sites within the CsSWEET2 protein sequence but also several phytohormone-responsive or stress-responsive *cis*-elements within the promoter region of *CsSWEET2* [19]. These results suggest that *CsSWEET2* may be phosphorylated or may bind to transcription factors involved in cold stress signalling pathways, such as the C-REPEAT BINDING FACTOR/COLD REGULATED(CBF-COR) regulatory signalling pathway [42]. These possible mechanisms through which *CsSWEET2* improves cucumber cold stress tolerance need to be further studied.

## 4. Materials and Methods

### 4.1. Plant Material and Growth Conditions

The northern China-type cucumber inbred line C49 was used in this study except where otherwise noted. Seedlings were grown in 9 cm × 9 cm plastic pots containing peat and vermiculite (2:1, *v/v*) in a phytotron under a temperature of 28 °C/18 °C (day/night) and a light intensity of 500 µmol quanta m^−2^ s^−1^ under a 16 h/8 h (day/night) photoperiod. Seedlings with 3–4 true leaves were subjected to different abiotic stress treatments. For high-salinity and drought treatments, 200 mM NaCl or 10% (*w/v*) polyethylene glycol (PEG) 6000 were dissolved in distilled water was applied to the roots. The plants were exposed to 40 °C for high-temperature treatment or 10 °C for cold treatment. The flooding treatment was conducted by submerging the roots in distilled water and keeping the peat-vermiculite saturated during the experiment. The second true leaves were harvested at 0 h, 1 h, 2 h, 3 h, 6 h, 9 h, and 12 h after high-salinity, drought, high-temperature, and flooding treatments were imposed. For cold treatment, the leaves were sampled at 0 h, 0.5 h, 1 h, 2 h, 4 h, 8 h, 12 h, 24 h, 48 h, and 72 h. All the collected samples were immediately frozen in liquid nitrogen and stored at −80 °C until use. Three independent trials were carried out for all the stress treatment experiments.

### 4.2. RNA Extraction and cDNA Synthesis

Total RNA was extracted from specified tissues using a Quick RNA Isolation Kit (Huayueyang, China) and treated with DNase I (Tiangen, Beijing, China) to remove any residual DNA. Single-stranded cDNA was synthesized using a PrimeScript^TM^ 1st Strand cDNA Synthesis Kit (Takara, Kyoto, Japan).

### 4.3. Cloning of CsSWEET2, Sequence Alignment and Phylogenetic Analysis

The full-length sequence of *CsSWEET2* was amplified using PrimeSTAR^®^ Max DNA Polymerase (Takara, Kyoto, Japan) under the following cycling conditions: 94 °C for 2 min; 30 cycles of 98 °C for 10 s, 55 °C for 5 s, and 72 °C for 1 min; and 72 °C for 10 min. The PCR products were subcloned into a *p*EASY-T1 vector (TransGen, Beijing, China) and sequenced. The primers used for *CsSWEET2* cloning are shown in Table S4.

Through BLAST analysis via the National Center for Biotechnology Information (https://www.ncbi.nlm.nih.gov (accessed on 2 December 2021)), The Arabidopsis Information Resource (TAIR) website (http://www.arabidopsis.org (accessed on 2 December 2021)), and the Cucurbit Genomics Database (http://cucurbitgenomics.org (accessed on 2 December 2021)) and using the sequence information of the CsSWEET2 protein, the amino acid sequences of related SWEET2 proteins in various plant species and of 17 Arabidopsis SWEET proteins (AtSWEET1 to AtSWEET17) were obtained (Table S3). Multiple sequence alignment and a phylogenetic tree were performed and constructed, respectively, as described previously [19].

### 4.4. Isolation of the Promoter Region of CsSWEET2 and GUS Expression Analysis

The putative promoter region of *CsSWEET2*, a 1910 bp PCR fragment upstream of the start codon (ATG), was amplified from cucumber genomic DNA using the primers listed in Table S4. The PCR product was digested with *Hind*III and *Xba*I and then cloned into the same sites of a pBI121 vector upstream of the *GUS* gene, yielding a *pCsSWEET2::GUS* construct. The *pCsSWEET2::GUS* plasmid was transferred into WT Arabidopsis plants (Col-0) using the *Agrobacterium tumefaciens* strain GV3101 and the floral dip method [43]. Transgenic seedlings were selected on half-strength Murashige and Skoog (1/2 MS) solid media supplemented with kanamycin (50 μg mL^−1^). The expression of the *GUS* gene in the transgenic plants was analysed by histochemical staining according to the methods described by Thomine et al. [44].

### 4.5. Subcellular Localization of CsSWEET2

The CsSWEET2-YFP fusion construct was generated using the Gateway-specific destination vector pX-YFP_GW [7] and the methods described by Ren et al. [25]. The ORF of *CsSWEET2* without a stop codon was amplified using specific primers harbouring the *attB1* and *attB2* sites (Table S4) and then cloned via a Gateway BP reaction into a pDONR221-f1 vector (Invitrogen, California, USA) followed by a Gateway LR reaction to insert the *CsSWEET2* sequence into the expression vector pX-YFP_GW, which is under the control of the CaMV 35S promoter. The CsSWEET2-YFP fusion construct was transiently expressed in the protoplasts of cucumber, rice, and Arabidopsis by PEG-mediated transformation as previously described [44,45,46]. The mCherry-labelled marker (TAIR stock No. CD3-959) was also expressed with CsSWEET2-YFP in protoplasts to indicate the endoplasmic reticulum position. Fluorescence signals were examined via a confocal laser-scanning microscope (Leica TCS SP8).

### 4.6. Functional Analysis of CsSWEET2 in Yeast

The ORF of *CsSWEET2* was first cloned into a pDONR221-f1 vector, and the construct was then transferred via the Gateway technique into the yeast expression vector pDRf1-GW [7], yielding a pDRf1-GW-CsSWEET2 construct. As a positive control, the *AtSWEET1* gene was also cloned into pDRf1-GW. The PCR primers used are listed in Table S4. The vector constructs or a pDRf1-GW empty vector (as a negative control) were separately transformed into the yeast mutant EBY.VW4000 using the lithium acetate method [47]. The transformed yeast cells were grown on selective synthetic deficient media without uracil (SD-Ura) supplemented with 2% (*w/v*) maltose as the sole carbon source.

To determine whether *CsSWEET2* transports hexose, complementation growth assays were performed as previously described [35]. The transformed yeast cells were grown in liquid SD-Ura media supplemented with 2% (*w/v*) maltose overnight until the optical density at 600 nm (OD_600_) of the cells reached 0.6. Serial dilutions (×1, ×10, ×100, and ×1000) were spotted onto solid SD-Ura media (pH 6.0) that included either 2% (*w/v*) maltose (as a control) or 2% (*w/v*) glucose and 2% (*w/v*) fructose constituting the sole carbon source. To determine the pH dependence of *CsSWEET2* activity, SD-Ura media were maintained at pH 4.0, 5.0, 6.0, and 7.0. For metabolic inhibitor treatment, 10 mM NH_4_Cl or 10 μM CCCP were added to the media. All the transformants were incubated at 30 °C for 3 days before being imaged.

### 4.7. qRT-PCR

qRT-PCR was performed using SYBR Premix Ex Tag^TM^ (Tli RNaseH Plus) (Takara, Kyoto, Japan) according to the manufacturer’s instructions on a LightCycler 480 system (Roche, Basel, Switzerland). Three biological replicates were included in the analysis. The cucumber *actin* gene was used as a reference gene, and the 2^−∆∆Ct^ method was used to calculate the relative transcript levels of *CsSWEET2* [48]. The primers used for qRT-PCR are listed in Table S4.

### 4.8. Ectopic Expression of CsSWEET2 in Arabidopsis

For overexpression vector construction, the ORF of *CsSWEET2* was amplified using specific primers (Table S4), digested with *BamH*I and *Sac*I, and then inserted into the same cut plasmid pBI121, which is driven by the CaMV 35S promoter. The overexpression construct was then introduced into *A. tumefaciens* strain GV3101, which was subsequently transformed into WT Arabidopsis (Col-0) plants as described previously [43].

For cold treatment, seeds from T_3_ homozygous lines were sown on solid 1/2 MS media supplemented with 1% (*w/v*) sucrose and 0.25% (*w/v*) gellan gum (Phytotech, Kansas, USA). The seeds were maintained at 4 °C for 3 days and then moved to a growth chamber, in which they were grown for 10 days at 22 °C (day and night); the light intensity was set to 125 μmol quanta m^−2^ s^−1^ under a 10 h light/14 h dark photoperiod (normal growth conditions). The Arabidopsis seedlings were then transplanted into soil (Sunshine^®^ Mix #1 Fafard^®^-1P, Sungro, Massachusetts, USA) and grown in the growth chamber under normal growth conditions for another 18 days. The plants were then subjected to cold treatment (4 °C) for 4 days, and whole shoots were collected for measurements of REL and sugar contents.

### 4.9. Measurements of REL and Sugar Contents

The REL of the WT and *CsSWEET2*-OE transgenic plants was determined using a previously described method [14], with minor modifications. Briefly, whole shoots were placed into tubes containing 10 mL of distilled water and shaken at 200 rpm for 2 h at 25 °C. The initial conductivity values of the samples and blank control (same amount of distilled water) were recorded as C1 and Cb1, respectively. The samples and blank control were then boiled for 30 min to induce electrolyte leakage, after which they were allowed to cool to room temperature. The electrolyte conductivities of the samples and blank control were recorded again as C2 and Cb2, respectively. The REL was then calculated as follows: Cr (%) = (C1 − Cb1)/(C2 − Cb2) × 100%. To measure the sugar contents, 0.2 g of fresh sample tissue was ground to a powder in liquid nitrogen and extracted in 2.0 mL of distilled water for 10 min at 95 °C. After cooling to room temperature, the extracts were centrifuged for 10 min at 8000 rpm. The supernatants were subsequently passed through a 0.45 µm filter and then used for measuring glucose, fructose, and sucrose contents via a high-performance liquid chromatograph (LC-10A VP, Shimadzu, Kyoto, Japan). All the samples were analysed in triplicate.

### 4.10. Statistical Analyses

For each treatment, the data presented are the means ± SEs of three independent biological replicates. Statistical analyses were performed with one-way ANOVA, and statistically significant differences were calculated using Duncan’s test at the 5% level (*p* < 0.05). Statistical analyses were conducted using SPSS statistical software (IBM, New York, NY, USA) and Excel software (Microsoft, Redmond, WA, USA).

## 5. Conclusions

In summary, *CsSWEET2*, which is highly expressed in the mesophyll tissue of cucumber leaves, encodes a hexose transporter that is located in the plasma membrane and endoplasmic reticulum. In addition, transgenic Arabidopsis plants overexpressing *CsSWEET2* have a significantly higher tolerance to cold stress than WT plants. Owing to its tropical origin and heat-loving habit, cucumber is extremely sensitive to cold, which can severely restrict cucumber plants from reaching their full genetic potential and can cause substantial yield and economic losses. Thus, increasing *CsSWEET2* activity via genetic engineering might constitute a promising new strategy for improving cucumber cold resistance and enhancing fruit yield and quality in the future.

## Data Availability

Not applicable.

## Supplementary Materials

The following supporting information can be downloaded at: https://www.mdpi.com/article/10.3390/ijms23073886/s1.
