# Supplementary material for: CsSWEET2, a Hexose Transporter from Cucumber (Cucumis sativus L.), Affects Sugar Metabolism and Improves Cold Tolerance in Arabidopsis
Source: Int J Mol Sci. 2022 Mar 31;23(7):3886. doi: 10.3390/ijms23073886 (PMC8999130; doi:10.3390/ijms23073886)
Supplement: Supplementary file 1 [file ijms-23-03886-s001.zip › Figure S1.pdf]

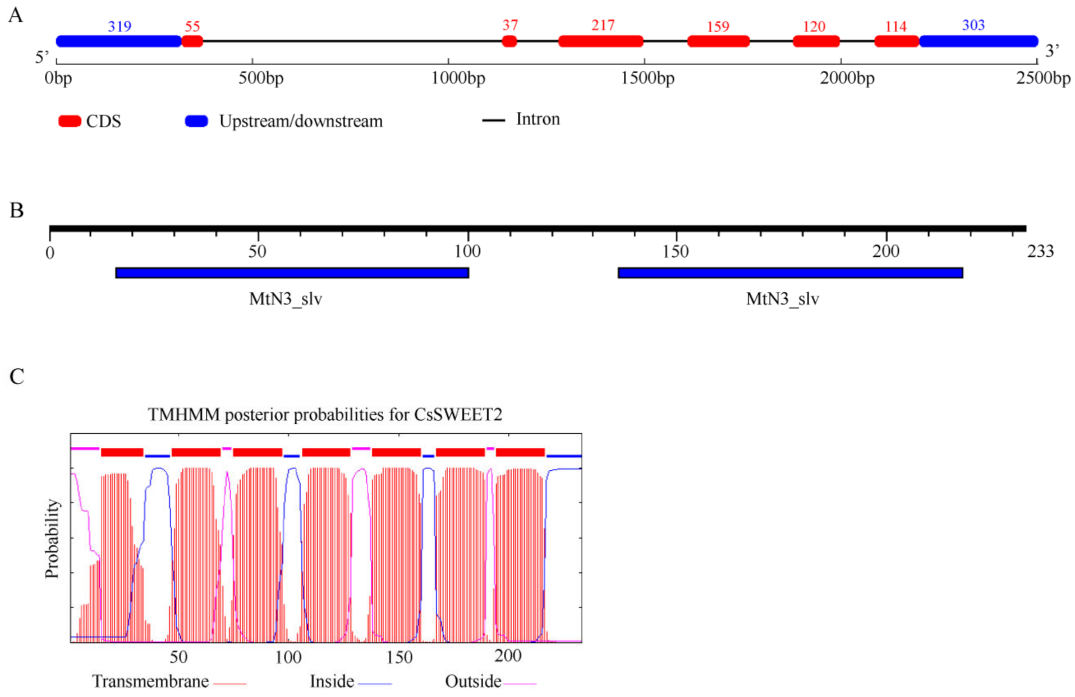

Figure S1. Structure, motif and transmembrane domain (TM) analysis of *CsSWEET2*. (A) The exon–intron structure of *CsSWEET2* was analysed using the web-based bioinformatics tool Gene Structure Display Server (GSDS, http://gsds.gao-lab.org). Exons and introns are shown as red boxes and black lines, respectively. Upstream and downstream untranslated regions are shown as blue boxes. The numbers above the boxes indicate the nucleotide numbers of the upstream and downstream untranslated regions, as well as each exon. Scales are shown at the bottom of the diagram. (B) Conserved motifs of CsSWEET2 were analysed by MOTIF Search (https://www.genome.jp/tools/motif). (C) TMs of the CsSWEET2 protein. The prediction website was TMHMM-2.0 (https://services.healthtech.dtu.dk/service.php?TMHMM-2.0).
