# Supplementary material for: CsSWEET2, a Hexose Transporter from Cucumber (Cucumis sativus L.), Affects Sugar Metabolism and Improves Cold Tolerance in Arabidopsis
Source: Int J Mol Sci. 2022 Mar 31;23(7):3886. doi: 10.3390/ijms23073886 (PMC8999130; doi:10.3390/ijms23073886)
Supplement: Supplementary file 1 [file ijms-23-03886-s001.zip › Figure S2.pdf]

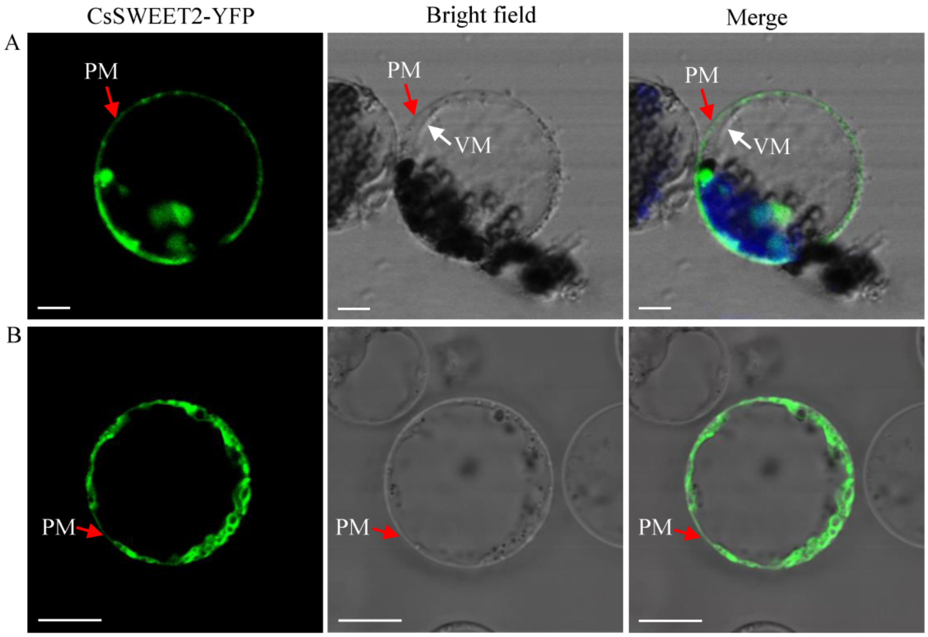

Figure S2. Subcellular localization of CsSWEET2 in cucumber and rice protoplasts. (A) Subcellular localization of CsSWEET2-YFP in cucumber mesophyll protoplasts. (B) Subcellular localization of CsSWEET2-YFP in chloroplast-free protoplasts of etiolated rice. The red or white arrows point to the plasma membranes or vacuolar membranes, respectively. The green signals indicate YFP, whereas the blue signals indicate chlorophyll auto fluorescence. Bright field images and merged images are also presented. PM, plasma membrane; VM, vacuolar membrane. Scale bars=10 μm.
