# Supplementary material for: CsSWEET2, a Hexose Transporter from Cucumber (Cucumis sativus L.), Affects Sugar Metabolism and Improves Cold Tolerance in Arabidopsis
Source: Int J Mol Sci. 2022 Mar 31;23(7):3886. doi: 10.3390/ijms23073886 (PMC8999130; doi:10.3390/ijms23073886)
Supplement: Supplementary file 1 [file ijms-23-03886-s001.zip › Figure S3.pdf]

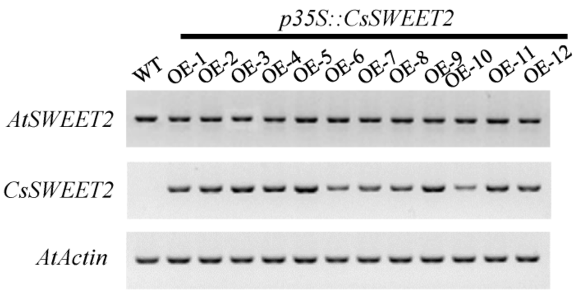

Figure S3. Reverse transcription PCR (RT-PCR) analysis of *CsSWEET2* and *AtSWEET2* expression in wild-type (WT) and transgenic Arabidopsis lines. The *CsSWEET2*-overexpressing (*CsSWEET2*-OE) lines, lines 5 and 9 (OE-5 and OE-9), which present much higher *CsSWEET2* transcript levels than the WT plants do and whose *AtSWEET2* expression is not affected, were selfed two times to generate homozygous lines.
