# Supplementary material for: CsSWEET2, a Hexose Transporter from Cucumber (Cucumis sativus L.), Affects Sugar Metabolism and Improves Cold Tolerance in Arabidopsis
Source: Int J Mol Sci. 2022 Mar 31;23(7):3886. doi: 10.3390/ijms23073886 (PMC8999130; doi:10.3390/ijms23073886)
Supplement: Supplementary file 1 [file ijms-23-03886-s001.zip › Figure S4.pdf]

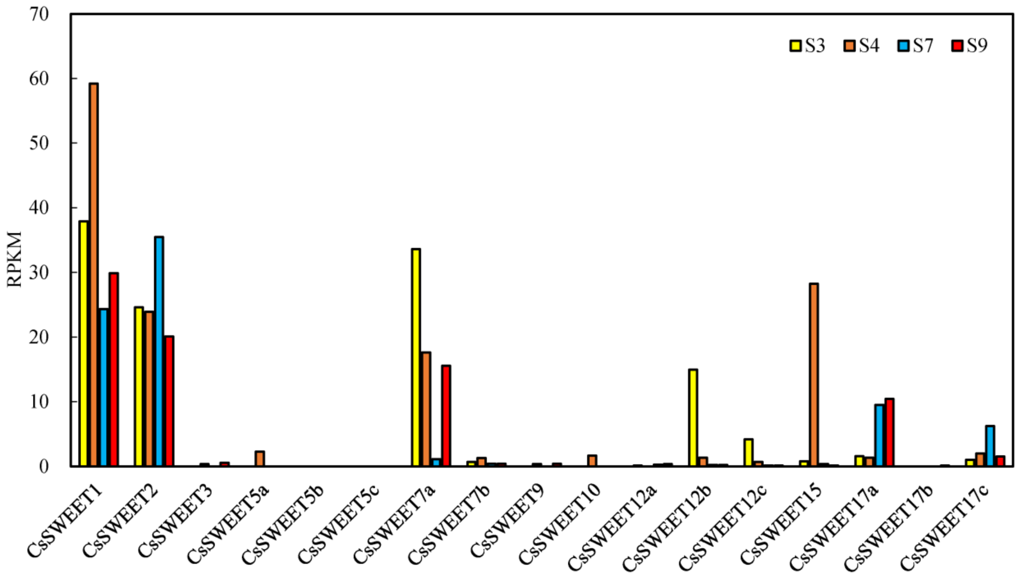

Figure S4. Expression analysis of 17 *CsSWEET* genes in different stages of cucumber leaves according to transcriptome data. Raw data were obtained from the Cucurbit Expression Atlas (http://cucurbitgenomics.org/rnaseq/home) under project PRJNA312872. The 17 cucumber *CsSWEET* genes were named according to their homologues in Arabidopsis and our earlier work [19]. RPKM, reads per kilobase per million mapped reads; S3, cotyledon of 4-week-old seedlings; S4, true leaf of 4-week-old seedlings; S7, young leaf of the second node from the top of cucumber plants; S9, mature leaf of the fifth node from the top of cucumber plants.
